# Evaluating GlicoPro Tear Substitute Derived from *Helix aspersa* Snail Mucus in Alleviating Severe Dry Eye Disease: A First-in-Human Study on Corneal Esthesiometry Recovery and Ocular Pain Relief

**DOI:** 10.3390/jcm13061618

**Published:** 2024-03-12

**Authors:** Antonio Ballesteros-Sánchez, José-María Sánchez-González, Giovanni Roberto Tedesco, Carlos Rocha-de-Lossada, Gianluca Murano, Antonio Spinelli, Cosimo Mazzotta, Davide Borroni

**Affiliations:** 1 Department of Physics of Condensed Matter, Optics Area, University of Seville, 41004 Seville, Spain; antbalsan@alum.us.es (A.B.-S.); jsanchez80@us.es (J.-M.S.-G.); 2 Department of Ophthalmology, Clínica Novovisión, 30008 Murcia, Spain; 3 Studio Oculistico Tedesco, 88025 Girifaldo, Italy; j.tedesco@libero.it; 4 Qvision Ophthalmology Department, VITHAS Almeria Hospital, 04120 Almeria, Spain; carlosrochadelossada5@gmail.com; 5 Ophthalmology Department, VITHAS Malaga, 29016 Malaga, Spain; 6 Regional University Hospital of Malaga, Hospital Civil Square, 29009 Malaga, Spain; 7 Department of Surgery, Ophthalmology Area, University of Seville, 41009 Seville, Spain; 8 Sacro Cuore—iGreco Ospedali Riuniti, 87100 Cosenza, Italy; 9 Biomeeting, 89124 Reggio Calabria, Italy; 10 Siena Crosslinking Center, 53035 Siena, Italy; cgmazzotta@libero.it; 11 Departament of Ophthalmology, Riga Stradins University, LV-1007 Riga, Latvia; 12 Centro Oculistico Borroni, 21013 Gallarate, Italy; 13 Eyemetagenomics Ltd., 71-75 Shelton Street, Covent Garden, London WC2 H9JK, UK

**Keywords:** *Helix aspersa*, ocular pain, corneal esthesiometry, tear substitute, dry eye disease

## Abstract

**Background:** To evaluate the effects of 10% GlicoPro tear substitute therapy in patients with severe dry eye disease (DED). **Methods:** In this prospective longitudinal study, 30 individuals receiving 10% GlicoPro four times daily for DED were evaluated. The ocular surface disease index (OSDI) questionnaire, average non-invasive break-up time (A-NIBUT), non-anesthetic and anesthetic corneal esthesiometry (CE), ocular pain, and the presence of conjunctivochalasis (CCH) were used as clinical endpoints. Treatment compliance using dosing diaries and AEs was assessed. **Results:** A significant improvement was observed in the clinical endpoints: the ΔOSDI questionnaire was −39.27 ± 13.22 [−65 to −15] points, ΔA-NIBUT was 3.10 ± 1.31 [1 to 5] s, Δnon-anesthetic CE was 14 ± 6.35 [5 to 25] mm, and Δanesthetic CE was 13 ± 5.35 [5 to 20] mm (*p* < 0.001 for all comparisons). Ocular pain was reduced in 92.5% of the patients at the end of the follow-up. However, there was no change in the presence of CCH. In addition, all the patients were fully compliant with the dosing and no AEs related to the use of the 10% GlicoPro tear substitute were reported. **Conclusions:** The 10% GlicoPro tear substitute has the potential to achieve beneficial effects in ocular surface treatments.

## 1. Introduction

Dry eye disease (DED), a chronic condition of the ocular surface, is prevalent in 5–50% of the global adult population and exhibits a higher incidence among women [1,2]. This multifactorial disease encompasses various elements, from demographic factors to environmental triggers and specific medical procedures, and it is characterized by a cascade of physiological changes, including tear film instability, elevated osmolarity, ocular inflammation, and neurosensory abnormalities [1,3]. These events favor the emergence of symptoms and signs that affect patients’ quality of life [4,5]. Additionally, DED is implicated in the dysfunction of both the innate and adaptive immune response systems [6,7], involving cellular components, such as monocytes, natural killer cells, and neutrophils, as well as molecular agents, such as pro-inflammatory cytokines and chemokines [3]. These entities collectively impair tear film stability and instigate a series of molecular and cellular events that exacerbate the condition, making its clinical management complex [3,8]. DED treatment often involves different strategies, ranging from basic solutions, such as tear substitutes, to more potent anti-inflammatory medications [9]. Despite these approaches, there remains a lack of therapies that can adequately address the heterogeneity of DED.

Mucins are critical biomolecules within this setting, providing essential functions, such as wettability, lubrication, and barrier formation on the ocular surface [10,11]. Their role extends to maintaining tear film quality and stability. In addition, alterations in mucin expression and secretion can escalate the vicious cycle of tear hyperosmolarity, and thus ocular surface inflammation [12,13]. Despite the key role mucins play in tear film dynamics and ocular surface health, a comprehensive understanding of their function and regulation remains unclear. Animal models have been instrumental in investigating DED. However, mucins are relatively poorly studied and characterized in this context [14]. There is considerable variation in the literature about the effects of DED on the structure and function of ocular mucins, which may be attributed to difficulties in collecting tear samples and differences in disease severity [11]. In addition, local inflammation may cause changes in mucin glycosylation, rather than affecting the mucin proteins themselves, highlighting the need for more precise research tools, such as genomic and proteomic studies [11]. Therefore, advancing the understanding of mucin biology in DED is essential to develop specific and effective therapeutic strategies.

Currently, new research is focusing on natural derivatives, such as GlicoPro, which is considered a promising agent for alleviating DED symptoms and signs. Obtained from the mucosa of the *Helix aspersa* snail, GlicoPro is a compound of various mucopolysaccharides, including glycosaminoglycans (GAGs) and sulfated GAGs (sGAGs) [15], which improves tear film stability by fortifying its mucinous layer and reducing ocular surface friction. GlicoPro also demonstrates regenerative, anti-inflammatory, and analgesic properties, as well as an impressive ability to bind to mucin, thanks to its thiomeric sGAGs, which form covalent bonds with mucin’s cysteine residues [15,16]. This allows for prolonged adherence on the corneal surface, potentially increasing its therapeutic effect [16]. However, to the best of our knowledge, there are no studies analyzing the effects of GlicoPro in humans.

Therefore, this study aims to evaluate the therapeutic effects of GlicoPro on patients with severe DED. Through this study, a comprehensive understanding of GlicoPro’s role in improving the quality of life and ocular health in this patient population is provided, enabling evidence-based decision making and guiding future research directions.

## 2. Materials and Methods

### 2.1. Study Design and Participants

This prospective, longitudinal study was carried out in Tedesco Eye Center (Girifalco, CZ, Italy), between November 2022 and February 2023. This study fulfilled the requirements of the Declaration of Helsinki and was approved by the internal review board (Approval Nr. 256-03/2022). Informed consent was obtained in all patients.

The inclusion criteria were as follows: (1) age > 65 y.o.; (2) DED diagnosis according to DEWS II [6]; and one of the following conditions: (2.1) ocular surface disease index (OSDI) score > 33 points; (2.2) non-invasive break-up time (NIBUT) < 10 s; and (3) meibomian gland dysfunction (MGD) according to the international workshop on MGD [17], meeting one of the following conditions: (3.1) eyelid margin or mucocutaneous junction irregularities; (3.2) eyelid margin vascularity; (3.3) capped or plugged Meibomian gland orifices; (3.4) atrophy of Meibomian glands; and (3.5) meibum quality and quantity decrease. The exclusion criteria included (1) degenerative diseases such as Parkinson’s disease or multiple sclerosis; (2) corneal infections and corneal dystrophies; (3) contact lens wearers; (4) active ocular allergy; (5) pregnant or lactating; and (6) patients who did not understand informed consent [18,19,20].

### 2.2. Treatment and Clinical Endpoints

Patients were instructed to apply 1 drop of 10% GlicoPro (Lacricomplex^®^, FB vision, Ascoli Piceno, AP, Italy) in each eye 4 times daily during a 12-week period. Clinical endpoint assessments were carried out in the sequence suggested by Ballesteros-Sanchez et al. [21] to best preserve the integrity of the tear film to avoid affecting test results: (1) OSDI questionnaire (expressed in points) [6,22]; (2) average NIBUT (A-NIBUT, expressed in seconds), which was measured with the Keratograph^®^ 5M (Oculus Optikgeräte GmbH, Wetzlar, Germany) [23]; and (3) non-anesthetic and anesthetic corneal esthesiometry (CE, expressed in millimeters), which was assessed with the Luneau Cochet-Bonnet aesthesiometer (Western Ophthalmics Corporation, Lynnwood, WA, USA) [24]. The presence of conjunctivochalasis (CCH) and ocular pain was also evaluated. Ocular or systemic adverse events (AEs) related to treatment were registered. Patient dosing diaries were used to assess compliance to treatment. Compliance was evaluated as the total number of doses that should have been administered multiplied by 100 [25]. All clinical endpoints were assessed by an ophthalmologist at screening, baseline (day 1), and 3 follow-up visits: (1) week 2 (15 ± 3 days), (2) week 4 (30 ± 5 days), and (3) week 12 (90 ± 10 days).

### 2.3. Statistical Analysis

SPSS statistics software version 28.0 (IBM Corporation, New York, NY, USA) was used for statistical analyses. GRANMO calculator, version 7.12 (Municipal Institute of Medical Research, Barcelona, Spain) was used for sample size estimation. It was calculated on the basis of assumed mean difference in A-NIBUT with a value of 6.31 ± 2.22 s. These assumed differences were based on the findings of a pilot study with 10 eyes of 10 patients. With these assumptions, a sample size of 24 eyes would yield a power >80% and a statistically significant paired difference of 95% confidence. In addition, this sample size was consistent with those provided by the TFOS DEWS II.

Continuous variables were displayed as the mean ± standard deviation (SD) with interquartile ranges [IQRs] [6,26], while ordinal categorical variables were expressed as frequencies (*n*) and percentages (%) [27]. Before analyses, one eye was randomly selected. An online randomizer program ‹https://www.randomization.com›(accessed on 1 March 2023) was used for the randomization scheme. After testing for normality with the Shapiro–Wilk test, the increment (Δ) was calculated to evaluate treatment efficacy. It was defined as the change from the baseline (B) to the last visit (LV) “Δ = LV − B” [28]. A repeated-measures ANOVA (parametric) or Friedman test (non-parametric) was performed to evaluate changes during follow-up. To determine the statistically significant differences between pairs of measures, a post hoc analysis with Bonferroni adjustment (parametric) or Wilcoxon signed ranks test (non-parametric) was performed [29]. For all comparisons, the level of significance was *p* < 0.05.

## 3. Results

A total of 30 eyes of 30 Caucasian patients, 11 (36.7%) men and 19 (63.3%) women with a mean age of 71.2 ± 13.3 (41–94) years, were recruited in this study. No loss to follow-up in the study was observed. The demographic characteristics are shown in Table 1.

### Clinical Endpoints Outcomes

**Figure 1:**
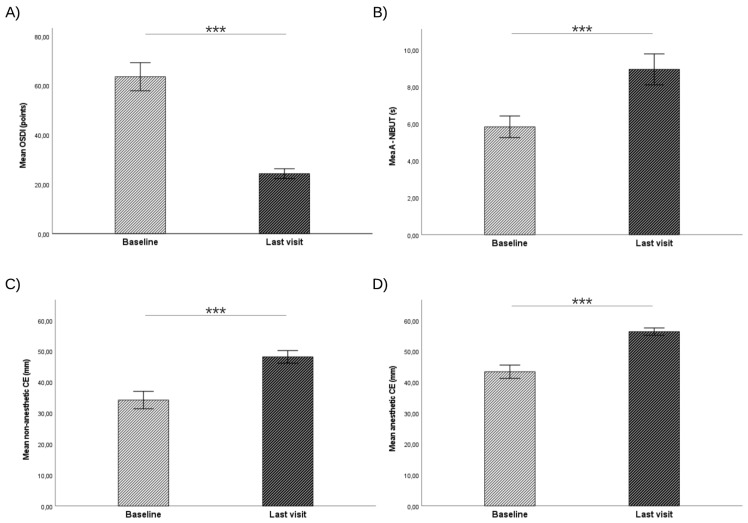
Bar graphs representing the mean values of clinical endpoints at baseline and last visit. The whiskers represent the 95% confidence interval (CI). (**A**) ocular surface disease index (OSDI) questionnaire. (**B**) average non-invasive tear film break-up time (A-NIBUT). (**C**) non-anesthetic corneal esthesiometry (CE). (**D**) anesthetic CE. *** *p*-value < 0.001.

The effectiveness of 10% GlicoPro tear substitutes in DED treatment is shown in Figure 1. 

After 12 weeks of follow-up, the patients reported a relevant improvement in ΔOSDI, ΔA-NIBUT, Δnon-anesthetic and Δanesthetic CE, with a mean value of −39.27 ± 13.22 [−65 to −15] points, 3.10 ± 1.31 [1 to 5] s, 14 ± 6.35 [5 to 25] mm, and 13 ± 5.35 [5 to 20] mm, respectively (*p* < 0.001 for all comparisons). The changes in DED symptoms and signs during the follow-up visits are shown in Table 2.

In the repeated measures analysis, all the clinical endpoints showed statistically significant differences (*p* < 0.001 for all comparisons). In addition, the post hoc analysis revealed that the non-anesthetic CE at baseline–non-anesthetic CE at 2 weeks (*p* = 0.157), and the anesthetic CE at baseline–anesthetic CE at 2 weeks (*p* = 0.705) were the only pairs of measures that showed no statistically significant differences. In addition, 92.5% of the patients reported no ocular pain at the end of the follow-up. However, there was no change at the end of the follow-up and exhibited no change in the presence of CCH. According to the doses recorded in the dosing diaries, all the patients were fully compliant with the dosing. In addition, no AEs were reported.

## 4. Discussion

The tear film hyperosmolarity is considered the trigger in the ocular surface inflammatory mechanism, resulting in DED symptoms and signs [3,30,31]. Tear substitutes are usually the first line of treatment for patients with DED [9,32]. New formulations that improve the tear film stability and restore the homeostasis of the ocular surface are under research [33,34]. The aim of this study was to analyze the effects of 10% GlicoPro tear substitute treatment in an elderly population with severe DED.

### 4.1. GlicoPro Efficacy

In this study, the ΔOSDI questionnaire, ΔA-NIBUT, Δnon-anesthetic and Δanesthetic CE, and ocular pain improved significantly after 12 weeks of follow-up. All the pairs of repeated measures showed statistically significant differences, except for the non-anesthetic CE at baseline–non-anesthetic CE at 2 weeks, and anesthetic CE at baseline–anesthetic CE at 2 weeks. The significant results in the ΔOSDI questionnaire and ΔA-NIBUT could be explained by the multiple effects of the 10% GlicoPro tear substitute treatment on the ocular surface. In an in vitro study, Mencucci et al. [15] aimed to evaluate the muco-adhesive, regenerative, anti-inflammatory, and analgesic properties of 10% GlicoPro tear substitute treatment in human corneal tissues induced with DED, comparing it to 0.15% sodium hyaluronate (SH) tear substitute treatment. Concerning the muco-adhesivity, a significantly higher percentage was observed in cells treated with 10% GlicoPro tear substitute compared to those treated with 0.15% SH tear substitute. Regarding the regenerative properties, the stratified squamous epithelium in the DED corneal tissues were regenerated by 24 h after 10% GlicoPro tear substitute treatment, while a significant loss of the cellular organization of this tissue was observed for 24 h with 0.15% SH tear substitute treatment. In addition, a clear restoration of corneal epithelium integrity was observed in the GlicoPro group by confocal microscopy. The 10% GlicoPro tear substitute treatment was also shown to reconstitute the basal levels of inflammatory cytokines, such as IL-1, IL-6, and IL-8, as well as ocular damage biomarkers, such as MMP-9 and MUC-4, compared to the 0.15% SH tear substitute treatment.

The mechanism by which CE is reduced in DED remains unclear. However, it is hypothesized that this reduction may be attributed to a decrease in the number of functionally intact corneal sub-basal nerve endings due to inflammation occurring in the anterior layers of the cornea. Several studies have reported results that are consistent with this theory. Benitez del Castillo et al. [34,35] and Villian et al. [36] reported decreased corneal sub-basal nerve counts in patients with DED. In addition, Cox et al. [37] showed that alterations in the corneal sub-basal nerves are more pronounced in aqueous-deficient dry eye (ADDE) than in evaporative dry eye (EDE). The mechanism of action of the 10% GlicoPro tear substitute treatment on CE could be explained by its effect on the suppression of inflammatory processes on the ocular surface [15]. As inflammation reduces and ocular surface integrity improves, functionally intact corneal sub-basal nerve endings may be stimulated more effectively [38]. In addition, our study has reported that this stimulation seems to occur progressively, achieving significant results after four weeks of follow-up.

Regarding ocular pain, it has been demonstrated that opiorphin in tears has potent analgesic properties [15,16]. Recently, Ozdogan et al. [39] reported that opiorphin levels are higher in patients with corneal foreign objects compared to healthy individuals. In addition, Mencucci et al. [15] reported that opiorphin concentration in the 10% GlicoPro tear substitute treatment is the same as that normally present in tears. Therefore, this endogenous pentapeptide may have therapeutic effects on neurosensory abnormalities responsible for the DED symptoms.

Overall, our findings suggest that the 10% GlicoPro tear substitute treatment has promising implications for DED treatment due to its unique properties. Therefore, further investigation of its mechanism of action may offer a solution for more effective DED therapies that reduce symptoms and signs for an extended period of time, thus improving patients’ quality of life.

### 4.2. GlicoPro Safety

In this study, no AEs were reported after application of the 10% GlicoPro tear substitute treatment. This lack of AEs can be attributed to its non-toxic nature, allowing for its administration in humans. In addition, the 10% GlicoPro tear substitute is preservative-free, which may substantially reduce the AEs associated with preservatives [40,41]. It is well-known that chronic exposure to tear substitute preservatives elevates concentrations of inflammatory markers in ocular tissues [42], leading to corneal epithelium and conjunctival goblet cell apoptosis [43,44,45]. Moreover, this preservative-induced cytotoxic effect on the ocular surface promotes clinical manifestations, such as conjunctival and corneal epithelial surface staining [46], which results in ocular discomforts including foreign body sensation, stinging, and burning [47].

### 4.3. Strengths and Limitations

This first-in-human study analyzed the effects of 10% GlicoPro tear substitute treatment in DED. However, many limitations need to be addressed. Firstly, an Index (OSDI) score of >33 points set to classify the participants as “severe’’ could also be indicative of neuropathic pain. A positive sign and a positive symptom should be present to classify a condition as a DED. The presence of only one could be due to other comorbidities or a pre-status to DED (not DED) [6,48,49]. The absence of a placebo group may influence the validity of the results, making it difficult for researchers to make strong claims about the efficacy and safety of 10% GlicoPro tear substitute treatment. In addition, despite the sample size calculation, the number of patients included may be small, leading to less accurate results. Furthermore, corneal fluorescein staining (CFS), which is usually the primary endpoint in DED studies, was not assessed in this study. However, this is due to the use of the Luneau Cochet-Bonnet aesthesiometer with anesthesia, which may increase corneal staining [50]. In addition, this instrument stimulates predominantly Aδ mechanosensitive nerve fibers and does not test the chemical and thermal sensitivity, which can be assessed by a non-contact gas aesthesiometer. Therefore, future studies incorporating a non-contact gas aesthesiometer and confocal microscopy analysis of corneal sub-basal nerve endings could better elucidate the effects of the 10% GlicoPro tear substitute treatment on CE. Although the study remains limited by sample configuration, there is, overall, a need for larger, well-designed, strictly blinded, randomized clinical trials evaluating the long-term effects of 10% GlicoPro tear substitute treatment.

## 5. Conclusions

The 10% GlicoPro tear substitute seems to achieve beneficial effects on DED symptoms and signs. This treatment significantly improves OSDI questionnaire scores, A-NIBUT, non-anesthetic and anesthetic CE, and ocular pain, with high compliance and no AEs after instillation. However, further studies at this concentration are warranted to validate our findings.

## Figures and Tables

**Table 1:** Baseline characteristics.

Demographics, Mean ± SD [IQR] or *n* (%)	*n* = 30
Age (years)	71.2 ± 13.3 (41–94)
Sex, male/female	11 (36.7)/19 (63.3)
Race, Caucasian	30 (100)
Related to DED, *n* (%)	
CCH	18 (60)
Ocular pain	30 (100)

CCH, Conjunctivochalasis; DED, dry eye disease; IQR, interquartile ranges, SD, standard deviation.

**Table 2:** Changes in clinical endpoints during follow-up visits.

Clinical Endpoints ^1^	Follow-Up				
	Baseline	2 Weeks	4 Weeks	12 Weeks	*p*-Value
OSDI, points	63.56 ± 15.28 [36.00–86.00]	51.80 ± 13.24 [31.00–77.00]	39.10 ± 11.10 [23.00–56.00]	24.30 ± 7.60 [20.00–50.00]	<0.001 *^,2^
A-NIBUT, s	5.83 ± 1.60 [3.20–8.00]	6.88 ± 1.92 [4.00–11.00]	8.00 ± 2.28 [4.50–13.00]	8.93 ± 2.24 [5.00–13.00]	<0.001 *^,3^
Non-anesthetic CE, mm	34.17 ± 7.55 [20.00–50.00]	34.83 ± 7.6 [20.00–50.00]	43.17 ± 6.22 [30.00–55.00]	48.17 ± 5.49 [35.00–55.00]	<0.001 *^,2^
Anesthetic CE, mm	43.33 ± 5.77 [35.00–55.00]	43.50 ± 5.89 [35.00–55.00]	50.67 ± 4.70 [40.00–60.00]	56.33 ± 3.20 [50.00–60.00]	<0.001 *^,2^

A-NIBUT, average non-invasive tear film break-up time; CE, corneal esthesiometry; mm, millimeters; OSDI, ocular surface disease index; s, seconds. ^1^ Expressed as mean ± standard deviation (SD) with interquartile ranges [IQRs]. ^2^ Parametric repeated measures ANOVA. ^3^ Non-parametric repeated measures ANOVA. * Statistical significance level of <0.05.

## Data Availability

The data presented in this study are available on request from the corresponding author (info.borroni@gmail.com).
